# Diversity of antimicrobial-resistant bacteria isolated from Australian chicken and pork meat

**DOI:** 10.3389/fmicb.2024.1347597

**Published:** 2024-02-19

**Authors:** Ojas V. A. Dixit, Mahboobeh Behruznia, Aidan L. Preuss, Claire L. O’Brien

**Affiliations:** ^1^Faculty of Science and Technology, University of Canberra, Canberra, ACT, Australia; ^2^School of Medicine, Science, Medicine and Health, University of Wollongong, Wollongong, NSW, Australia

**Keywords:** antibiotic, antibiotic resistance, antimicrobial resistance, antimicrobial susceptibility testing, multidrug-resistance, resistance genes, whole-genome sequencing

## Abstract

Antimicrobial-resistant bacteria are frequently isolated from retail meat and may infect humans. To determine the diversity of antimicrobial-resistant bacteria in Australian retail meat, bacteria were cultured on selective media from raw chicken (*n* = 244) and pork (*n* = 160) meat samples obtained from all four major supermarket chains in the ACT/NSW, Australia, between March and June 2021. Antimicrobial susceptibility testing (AST) was performed for 13 critically and 4 highly important antibiotics as categorised by the World Health Organization (WHO) for a wide range of species detected in the meat samples. A total of 288 isolates underwent whole-genome sequencing (WGS) to identify the presence of antimicrobial resistance (AMR) genes, virulence genes, and plasmids. AST testing revealed that 35/288 (12%) of the isolates were found to be multidrug-resistant (MDR). Using WGS data, 232/288 (81%) of the isolates were found to harbour resistance genes for critically or highly important antibiotics. This study reveals a greater diversity of AMR genes in bacteria isolated from retail meat in Australia than previous studies have shown, emphasising the importance of monitoring AMR in not only foodborne pathogenic bacteria, but other species that are capable of transferring AMR genes to pathogenic bacteria.

## Introduction

The prevalence of antimicrobial-resistant (AMR) bacteria and resistance to traditional antibiotics is increasing globally and is therefore a significant global health issue (Collignon, 2015). Antimicrobials are used to prevent and control bacterial infections in food and animal production systems; however, their overuse in the agri-food industry has expedited the spread of AMR bacteria worldwide. The use of antimicrobials in food animal production selects for AMR bacteria, which may be transmitted to humans via zoonotic bacteria in the food chain (Barlow et al., 2015). The continued prophylactic use of antimicrobials in the Australian meat industry no doubt contributes to the acquisition and maintenance of AMR (Landers et al., 2012; Kirchhelle, 2018).

European Union legislation imposed in 2022 prohibits the routine use and prophylactic use of antimicrobial medicinal products in farming, including the use of medicated feeds.^1^ The United States has followed a similar path; in 2019, approximately 60% of broilers were raised in no antibiotics ever (NAE) conditions.^2^ Australian government regulations do not go as far, as the prophylactic use of antimicrobials is still allowed. In 2015, Australia was reported to have relatively low rates of antibiotic resistance to third-generation cephalosporins, fluoroquinolones, aminoglycosides, and carbapenems (Collignon, 2015). However, a recent study showed that AMR rates are variable across Australia, with some areas showing high rates of AMR in hospital-acquired pathogens. It was estimated that 1,031 human deaths were attributed to five hospital-associated AMR pathogens in 2020 (Wozniak et al., 2022). This estimate is four times higher than an estimate provided by the OECD in 2018 (Dunachie et al., 2020).

Carbapenems are useful antibiotics because of their broad spectrum of activity and effectiveness against both Gram-positive and Gram-negative bacteria (Papp-Wallace et al., 2011). Colistin, a last resort antimicrobial, is used to treat carbapenem-resistant *Enterobacteriaceae* (CRE) infections in many countries; however, colistin resistance has emerged in CRE, producing conditions for which no effective antibiotic treatment is now available (antimicrobial resistance, El-Sayed Ahmed et al., 2020; WHO, 2020). Colistin is also used to treat infections caused by other MDR bacteria, including *Pseudomonas aeruginosa* and *Acinetobacter baumannii*; however, colistin resistance has emerged in these species as well. Some bacteria, such as *Serratia* spp., *Proteus* spp., and *Burkholderia* spp., are naturally resistant to colistin (Aghapour et al., 2019); however, they may still acquire plasmids with colistin resistance genes and therefore still participate in the spread of colistin resistance via horizontal gene transfer (Zhong et al., 2022). Very few studies have assessed the presence of colistin resistance genes in non-pathogenic species of bacteria; however, such species may act as reservoirs for colistin resistance.

Bacterial species (spp.), such as *Campylobacter* spp. (Habib et al., 2020), *Escherichia coli* (Vangchhia et al., 2018; Touchon et al., 2020; Abraham et al., 2020), *Enterococcus* spp. (Lee et al., 2021), and *Salmonella* spp. (Abraham et al., 2020), are known to be pathogenic. These species are frequently used as AMR “indicators” in surveillance studies of production animals because they are important in human disease, are relatively easy to culture and identify, and have known AMR minimum inhibitory concentrations (MIC) (Cameron and McAllister, 2016). While pathogenic bacteria typically contain AMR genes, other species of bacteria and bacteriophages are capable of transferring MGEs to pathogenic bacteria, but are often overlooked in surveillance studies because they are not pathogenic.

Many AMR studies have revealed *Campylobacter* spp., *Escherichia* spp., *Salmonella* spp., and *Enterococcus* spp. to be widespread in meat samples. *E. coli* is a common member of the enteric community of poultry and other birds (Blyton et al., 2015). The poultry sector has been identified as a likely source of extended-spectrum β-lactamase (ESBL)-producing Gram-negative bacteria that can infect people who consume or handle contaminated meat (Leverstein-van Hall et al., 2011). According to Overdevest et al. (2011), 80% of ESBL genes found in chickens are mostly identical to ESBL genes found in human rectal swabs, and *E. coli* typing confirmed the similarity between chicken and human strains, albeit using low-resolution typing methods (Kluytmans et al., 2013).

AMR bacteria are of serious concern because they pose a direct threat to humans. Screening for the presence of AMR bacteria in meat produced for human consumption, beyond the most common foodborne pathogens, may provide important information about the diversity of AMR genes and the bacteria that carry them in food-producing animals. Additionally, it is important to know the extent to which AMR genes are encoded on MGEs, as they may be transferred to pathogenic bacteria from bacteria not commonly screened in surveillance studies. The potential virulence of strains isolated from retail meat is also not commonly assessed. Therefore, the goals of this study were to isolate and identify bacterial species beyond the commonly surveyed food pathogens in Australian retail chicken and pork meat using selective media and whole-genome sequencing; to assess the extent of phenotypic AMR; and to identify MGEs and virulence genes present in the bacteria to understand their ability to disseminate AMR genes and cause disease.

## Materials and methods

### Sample acquisition and processing

A total of 404 meat samples (244 chicken and 160 pork) were purchased by a third-party contractor from Aldi (39 chicken, 39 pork), Coles (85 chicken, 41 pork), IGA (41 chicken, 33 pork), and Woolworths (79 chicken, 47 pork) supermarkets across 39.5/50 ACT/NSW electorates in Australia between March and June 2021. All chicken and pork meat samples available at each supermarket were purchased, provided they met the inclusion criteria: raw, unprocessed, unmarinated, unseasoned, and not labelled either “free range” or “organic.” Once purchased, all meat samples were transported, stored at 4°C, and processed within 24 h, before their expiration date. All sample packaging was disinfected with 80% ethanol before being processed aseptically in a Class II Biosafety Cabinet. Approximately 10 g of meat was taken from four locations of each sample and added to both 25 mL pre-warmed peptone buffered water and 25 mL Bolton broth (for *Campylobacter* isolation) and homogenised using a stomacher. Approximately 20 mL of homogenate for chicken samples obtained from a single supermarket were combined in a single tube. The same was done to combine pork samples from a single supermarket. This resulted in a total of 302 pooled samples (152 chicken, 150 pork). Of the pooled samples, 211 (70%) samples comprised a single brand product, 82 (27%) comprised two, seven (2%) comprised three, and two comprised four (1%). These pooled samples were grown in selective media.

### Selection of isolates

The selective media used to grow bacteria from the meat samples included Brilliance™ ESBL agar, used for the detection of ESBL-producing bacteria; Brilliance™ CRE agar, used for the detection of carbapenem-resistant Enterobacteriaceae (CRE); Brilliance™ VRE agar, used for the detection of vancomycin-resistant enterococci (VRE); *Campylobacter* selective agar (CAMPY), used for the selection of *Campylobacter* spp.; MacConkey (MAC) agar, used for the identification and differentiation of *Enterobacteriaceae* spp., including *E. coli*; and xylose lysine deoxycholate (XLD) agar, used for the identification of *Salmonella* spp. A 1 mL aliquot of the PBW homogenate sample was added to selenite broth at 41°C for 18 h with shaking to select for *Salmonella*. Plating on XLD agar at 37°C overnight followed. A representative of each different colony, based on colony morphology and colour, was selected for each media type, regardless of whether they appeared to be a target organism for the selective agar or not. A freezer stock containing 30% glycerol was made for each isolate. Whole-genome sequencing (WGS) was performed for 288 isolates, with all isolates that grew on Brilliance™ ESBL, Brilliance™ CRE, Brilliance™ VRE, and CAMPY agar being prioritised, and the remainder being made up of isolates that grew on MAC or XLD agar. A single isolate of *E. coli* was randomly chosen from each electorate, despite having identified multiple different isolates of *E. coli* for each electorate. Due to the small number of isolates grown on XLD, MAC and XLD results are presented together.

### Antimicrobial susceptibility testing

Antimicrobial sensitivity testing was performed for the 288 isolates using an automated MIC broth microdilution method and commercially prepared Gram-negative (CMV3AGNF™) and *Campylobacter* spp. (EUCAMP2™) Sensititre™ antibiotic plates (Thermo Scientific™). All bacterial isolates, apart from *Campylobacter* spp., were grown from glycerol freezer stocks on their respective agar (Brilliance™ ESBL/Brilliance™ CRE/Brilliance™ VRE, MAC, and XLD) and incubated overnight at 37°C. The *Campylobacter* isolates were grown on CAMPY agar and incubated at 41°C for 48 h in anaerobic jars with CampyGen sachets (Oxoid™).

After incubation, a few colonies from each agar plate were transferred to 5 mL Sensititre™ demineralised sterile water (Thermo Scientific™) to achieve a density equivalent to the 0.5 McFarland standard. A 10 μL aliquot of each 0.5 density dilution was transferred to a 5 mL Sensititre™ Mueller Hinton Broth and mixed well. A Sensititre™ 96-well plate was then inoculated with 50 μL volume per well of the suspension using the Sensititre™ AIM™ (Automated Inoculation Delivery) system. The Gram-negative CMV3AGNF™ plates were sealed and incubated at 37°C in a non-CO_2_ incubator for 24 h, and at 41°C for 48 h for the EUCAMP2™ plates. Following incubation, plates were placed inside a Sensititre™ Vizion™ Digital MIC viewing system, and results were recorded and interpreted using Sensititre™ SWIN™ software, based on the Clinical & Laboratory Standards Institute (CLSI) breakpoints for MIC determination.

To determine whether or not an isolate was MDR, we used the definitions as set out by Magiorakos et al. (2012). If a species was not included in this definition, then we used the same definition as a species from the same genus; if no species or genus encountered was included in their definition, then we searched the literature to determine if the genus/species was intrinsically resistant to the antibiotics tested. As with the Magiorakos et al. (2012) definition, intrinsic resistance was not taken into account.

### Whole-genome sequencing and analysis

DNA from the 288 prioritised isolates was extracted from a 1 mL aliquot of an overnight broth culture using Bioline^®^ ISOLATE II Genomic DNA Kits according to the manufacturer’s protocol. Quantification of DNA was performed using a TapeStation system (Agilent Technologies, Inc.). plexWell™ 96 Kits (seqWell™) were used for library preparation, and sequencing was performed on an Illumina^®^ NovaSeq™ platform (Illumina^®^, Inc.) in a 150 bp paired-end format.

The raw paired-read data of each isolate were assembled using the St. Petersburg genome assembler (SPAdes) (Bankevich et al., 2012) tool from the Bacterial and Viral Bioinformatics Resource Center (BV-BRC) (Olson et al., 2023). The assembled sequences were annotated using the Rapid Annotations utilising Subsystems Technology (RASTtk) (Brettin et al., 2015) tool kit based on genus/species identification. Each assembled sequence was given a taxonomy-based annotation (genus or species) using the NCBI’s BLAST tool. The acquired antibiotic resistance genes, plasmids, and virulence genes were identified using the Mobile Genetic Element (MGE) finder tool from the Center for Genomic Epidemiology (CGE). The MGE tool identifies mobile genetic elements and their relation to AMR genes and virulence factors (Johansson et al., 2021). The PathogenFinder 1.1 tool, also from the CGE, was used to predict the likelihood of isolates being pathogenic to humans (Cosentino et al., 2013). Multilocus sequence typing was performed using the MLST tool from CGE, which can identify the sequence types (ST) of 66 bacterial species (Larsen et al., 2012).

## Results

**Figure 1:**
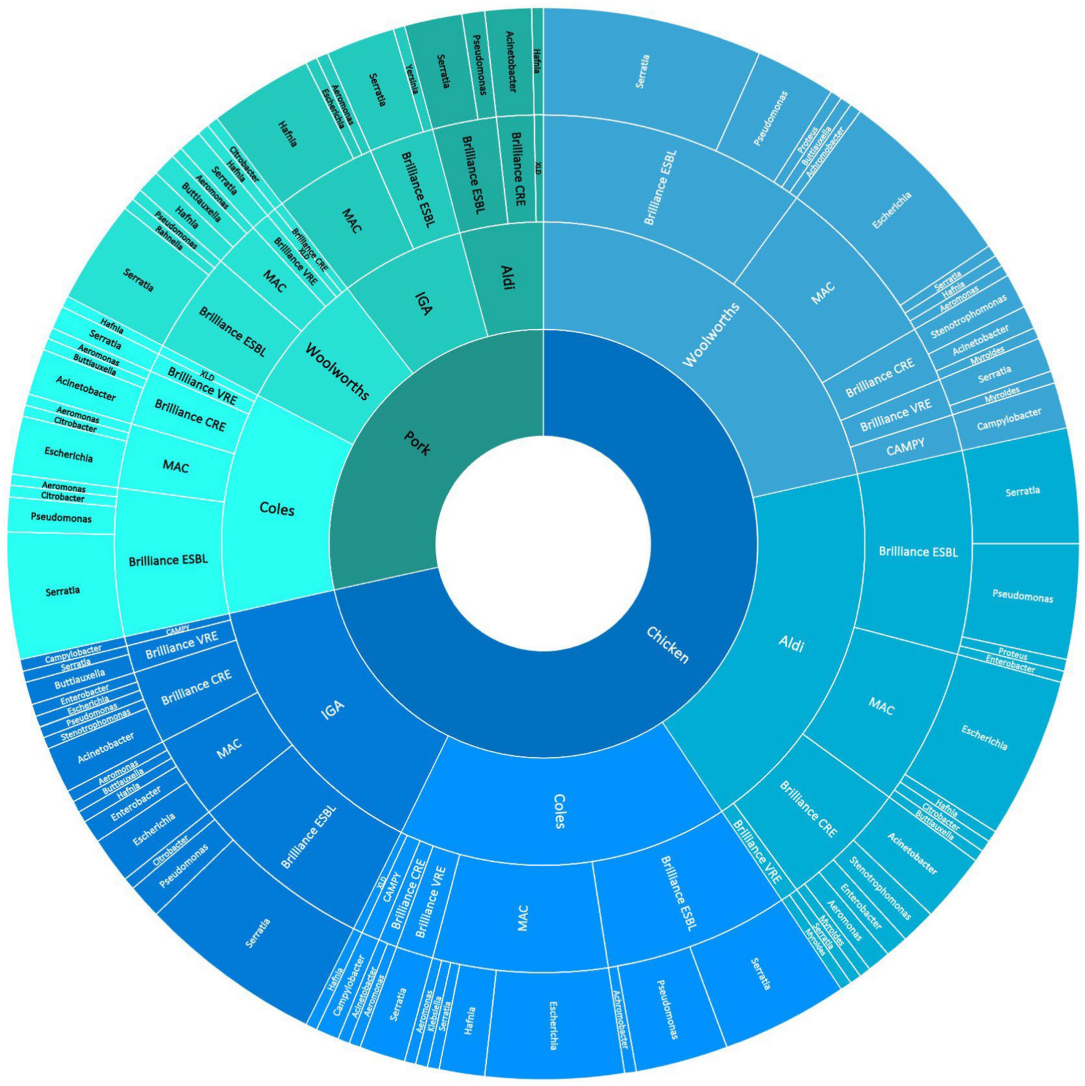
Sunburst diagram shows the breakdown of meat samples (innermost ring) across supermarket chains from 39.5 electorates in the ACT/NSW regions of Australia (second innermost ring), the abundance of isolates grown on various selective media (third innermost ring), and the abundance of bacterial genera grown on the media (outermost ring).

The breakdown of bacterial genera detected according to the selective media used for and the supermarket chain from which the meat samples were purchased for pooled chicken and pork samples is presented in Figure 1. For the pooled chicken samples, *Serratia* spp. were most commonly isolated (67/206, 32%), followed by *E. coli* (47/206, 23%), *Pseudomonas* spp. (29/206, 14%), and *Acinetobacter* spp. (13/206, 6%). For the pooled pork samples, *Serratia* spp. were most commonly isolated (35/82, 43%), followed by *Hafnia* spp. (14/82, 17%), *Acinetobacter* spp. (8/82, 9%), and *E. coli* (6/82, 7%). Overall, the 288 isolates represented 17 different genera (Table 1). A total of 41 isolates produced colonies on Brilliance™ CRE agar (30 chicken, 11 pork), 17 on Brilliance™ VRE agar (13 chicken, 4 pork), 132 on Brilliance™ ESBL agar (91 chicken, 41 pork), 7 on CAMPY agar (7 chicken, 0 pork), and 91 on MAC/XLD agar (65 chicken, 26 pork). None of the isolates that produced colonies on Brilliance™ VRE agar and were presumed to be *Enterococcus*, according to WGS identification, were indeed *Enterococcus*. All isolates from Brilliance™ VRE were Gram-negative bacteria, which vancomycin is not active against. None of the isolates that grew in selenite broth, and later on XLD, were *Salmonella*. All isolates from XLD belonged to the closely related genus *Hafnia*.

**Table 1:** Frequency of the 17 bacterial genera isolated from all pooled chicken and pork samples.

Organisms	Chicken	Pork
	Observed (*n* = 206, 71.53%)	Observed (*n* = 82, 28.47%)
*Achromobacter* spp.	2 (1.0%)	0 (0.0%)
*Acinetobacter* spp.	13 (6.3%)	8 (9.8%)
*Aeromonas* spp.	6 (2.9%)	5 (6.1%)
*Buttiauxella* spp.	5 (2.4%)	3 (3.7%)
*Campylobacter* spp.	7 (3.4%)	0 (0.0%)
*Citrobacter* spp.	2 (1.0%)	3 (3.7%)
*Enterobacter* spp.	6 (2.9%)	0 (0.0%)
*Escherichia* spp.	47 (22.8%)	6 (7.3%)
*Hafnia* spp.	8 (3.9%)	14 (17.1%)
*Klebsiella* spp.	1 (0.5%)	0 (0.0%)
*Myroides* spp.	4 (1.9%)	0 (0.0%)
*Proteus* spp.	2 (1.0%)	0 (0.0%)
*Pseudomonas* spp.	29 (14.1%)	6 (7.3%)
*Rahnella* spp.	0 (0.0%)	1 (1.2%)
*Serratia* spp.	67 (32.5%)	35 (42.7)
*Stenotrophomonas* spp.	7 (3.4%)	0 (0.0%)
*Yersinia* spp.	0 (0.0%)	1 (1.2%)

### Antimicrobial resistance phenotyping

The 288 isolates that underwent WGS were tested for antibiotic sensitivity using an automated minimum inhibitory concentration (MIC) broth microdilution method and commercially available Gram-negative (CMV3AGNF) and *Campylobacter* (EUCAMP2) Sensititre™ antibiotic plates (Thermo Scientific™). According to World Health Organization (2022), each antibiotic on the list is either a critically important antibiotic (CIA) or a highly important antibiotic (HIA) for human health. Based on the chosen antibiotics, each isolate was evaluated to determine whether it was MDR, XDR, or PDR.

The AMR phenotype and MDR results for all 288 isolates that underwent WGS are provided in Table 2, for all pooled chicken and pork samples across all selective media used in the study. Of the 288 isolates, 35 (12%) were MDR, and of these, 17 were *Serratia* spp. that grew on Brilliance™ ESBL (15 chicken, 2 pork). The MDR criteria did not include antibiotics for which *Serratia* spp. are intrinsically resistant. The remaining 18 MDR isolates belonged to a variety of bacterial genera, including *Proteus* spp. (2/18, 11%), *Rahnella* spp. (1/18, 6%), *Yersinia* spp. (1/18, 6%), *Buttiauxella* spp. (2/18, 11%), *Citrobacter* spp. (2/18, 11%), *Aeromonas* spp. (3/18, 17%), *Acinetobacter* spp. (1/18, 6%), *Enterobacter* spp. (4/18, 22%), *Pseudomonas* spp. (1/18, 6%), and *Escherichia* spp. (1/18, 6%). All of the MDR bacteria were isolated from Brilliance™ ESBL (25/35, 71%), Brilliance™ CRE (9/35, 26%), or Brilliance™ VRE agar (1/35, 3%). No MDR isolate was cultured from either MAC or XLD. The frequency of MDR varied across bacterial isolates from chicken and pork samples and across supermarkets, with 28% (11/39), 1% (1/85), 15% (6/41), and 13% (10/79) of chicken isolates; and 3% (1/39), 10% (4/41), 0% (0/33), and 4% (2/47) of pork isolates being MDR from Aldi, Coles, IGA, and Woolworths, respectively.

**Table 2:** Antibiotic resistance phenotype and multiple drug resistance results for bacterial genera isolated from various media for all pooled chicken and pork samples.

Media	Meat sample	Bacterial genus	AMP	AUG2	AXO	AZI	CHL	CIP	ERY	FIS	FOX	GEN	NAL	STR	SXT	TET	XNL	MDR *n* (%)
			1–32 mg/mL	0.5/1.16–32 mg/mL	0.25–64 mg/mL	0.12–16 mg/mL	2–32 mg/mL	0.015–4 mg/mL	1–128 mg/mL	16–256 mg/mL	0.5–32 mg/mL	0.25–16 mg/mL	0.5–32 mg/mL	2–64 mg/mL	2–64 mg/mL	4–32 mg/mL	0.12–8 mg/mL	
Brilliance™ ESBL	Chicken (*n* = 91)	*Achromobacter* spp., *n* = 2 (%)	0 (0)	0 (0)	0 (0)	0 (0)	1 (50)	0 (0)	0 (0)	0 (0)	0 (0)	0 (0)	0 (0)	0 (0)	0 (0)	0 (0)	0 (0)	**0 (0)**
		*Buttiauxella* spp., *n* = 1 (%)	1 (100)	1 (100)	1 (100)	0 (0)	1 (100)	0 (0)	0 (0)	0 (0)	1 (100)	0 (0)	0 (0)	0 (0)	1 (100)	1 (100)	0 (0)	**1 (100)**
		*Citrobacter* spp., *n* = 1 (%)	1 (100)	1 (100)	1 (100)	0 (0)	1 (100)	0 (0)	0 (0)	0 (0)	1 (100)	0 (0)	0 (0)	0 (0)	0 (0)	0 (0)	0 (0)	**1 (100)**
		*Enterobacter* spp., *n* = 1 (%)	1 (100)	1 (100)	1 (100)	0 (0)	1 (100)	0 (0)	0 (0)	0 (0)	1 (100)	0 (0)	0 (0)	0 (0)	1 (100)	1 (100)	0 (0)	**1 (100)**
		*Proteus* spp., *n* = 2 (%)	1 (100)	1 (50)	2 (100)	0 (0)	2 (100)	0 (0)	0 (0)	0 (0)	0 (0)	1 (50)	0 (0)	1 (50)	0 (0)	1 (50)	0 (0)	**2 (100)**
		*Pseudomonas* spp., *n* = 28 (%)	0 (0)	0 (0)	3 (10.7)	0 (0)	25 (89.3)	1 (3.5)	0 (0)	0 (0)	0 (0)	0 (0)	1 (3.5)	1 (3.5)	16 (57.4)	0 (0)	1 (3.5)	**0 (0)**
		*Serratia* spp., *n* = 56 (%)	52 (92.8)	42 (75)	46 (82.1)	0 (0)	12 (21.4)	0 (0)	0 (0)	0 (0)	32 (57.1)	1 (1.8)	5 (8.9)	4 (7.1)	6 (10.7)	9 (16.1)	1 (1.8)	**15 (27)**
	Pork (*n* = 41)	*Aeromonas* spp., *n* = 1 (%)	0 (0)	0 (0)	0 (0)	0 (0)	0 (0)	0 (0)	0 (0)	0 (0)	1 (100)	0 (0)	0 (0)	0 (0)	0 (0)	1 (100)	0 (0)	**0 (0)**
		*Citrobacter* spp., *n* = 1 (%)	1 (100)	1 (100)	0 (0)	0 (0)	0 (0)	0 (0)	0 (0)	0 (0)	1 (100)	0 (0)	0 (0)	0 (0)	1 (100)	1 (100)	0 (0)	**1 (100)**
		*Pseudomonas* spp., *n* = 6 (%)	0 (0)	0 (0)	2 (33.3)	0 (0)	2 (33.3)	2 (33.3)	0 (0)	0 (0)	0 (0)	0 (0)	0 (0)	0 (0)	4 (66.6)	2 (33.3)	0 (0)	**0 (0)**
		*Rahnella* spp., *n* = 1 (%)	1 (100)	1 (100)	1 (100)	0 (0)	0 (0)	0 (0)	0 (0)	0 (0)	1 (100)	0 (0)	0 (0)	0 (0)	0 (0)	0 (0)	0 (0)	**1 (100)**
		*Serratia* spp., *n* = 31 (%)	25 (80.6)	21 (67.7)	25 (80.6)	0 (0)	0 (0)	1 (3.2)	0 (0)	0 (0)	18 (58.1)	0 (0)	2 (6.4)	2 (6.4)	0 (0)	2 (6.4)	2 (6.4)	**2 (6.5)**
		*Yersinia* spp., *n* = 1 (%)	1 (100)	1 (100)	1 (100)	0 (0)	0 (0)	0 (0)	0 (0)	0 (0)	1 (100)	0 (0)	0 (0)	0 (0)	0 (0)	0 (0)	0 (0)	**1 (100)**
Brilliance™ CRE	Chicken (*n* = 30)	*Acinetobacter* spp., *n* = 13 (%)	0 (0)	0 (0)	5 (38.5)	0 (0)	0 (0)	2 (15.4)	0 (0)	0 (0)	1 (7.7)	3 (23.1)	0 (0)	2 (15.4)	1 (7.7)	0 (0)	1 (7.7)	**1 (7.7)**
		*Aeromonas* spp., *n* = 3 (%)	0 (0)	0 (0)	1 (33.3)	1 (33.3)	1 (33.3)	0 (0)	0 (0)	0 (0)	1 (33.3)	2 (66.7)	0 (0)	1 (33.3)	0 (0)	3 (100)	1 (33.3)	**2 (66.7)**
		*Enterobacter* spp., *n* = 3 (%)	3 (100)	3 (100)	1 (33.3)	0 (0)	0 (0)	1 (33.3)	0 (0)	0 (0)	3 (100)	1 (33.3)	0 (0)	1 (33.3)	1 (33.3)	1 (33.3)	0 (0)	**3 (100)**
		*Escherichia* spp., *n* = 1 (%)	0 (0)	0 (0)	1 (100)	1 (100)	0 (0)	1 (100)	0 (0)	1 (100)	0 (0)	0 (0)	1 (100)	1 (100)	0 (0)	0 (0)	1 (100)	**1 (100)**
		*Myroides* spp., *n* = 2 (%)	0 (0)	0 (0)	0 (0)	0 (0)	0 (0)	1 (50)	0 (0)	0 (0)	0 (0)	1 (50)	0 (0)	0 (0)	0 (0)	0 (0)	0 (0)	**0 (0)**
		*Pseudomonas* spp., *n* = 1 (%)	0 (0)	0 (0)	1 (100)	0 (0)	1 (100)	1 (100)	0 (0)	0 (0)	0 (0)	1 (100)	0 (0)	1 (100)	0 (0)	0 (0)	0 (0)	**1 (100)**
		*Stenotrophomonas* spp., *n* = 7 (%)	0 (0)	0 (0)	0 (0)	0 (0)	3 (42.9)	0 (0)	0 (0)	0 (0)	0 (0)	0 (0)	0 (0)	0 (0)	3 (42.9)	0 (0)	0 (0)	**0 (0)**
	Pork (*n* = 11)	*Acinetobacter* spp., *n* = 8 (%)	0 (0)	0 (0)	1 (12.5)	0 (0)	1 (14.3)	4 (62.5)	0 (0)	0 (0)	0 (0)	2 (37.5)	0 (0)	0 (0)	0 (0)	0 (0)	0 (0)	**0 (0)**
		*Aeromonas* spp., *n* = 1 (%)	0 (0)	0 (0)	1 (100)	0 (0)	1 (100)	1 (100)	0 (0)	0 (0)	1 (100)	1 (100)	0 (0)	0 (0)	1 (100)	1 (100)	0 (0)	**1 (100)**
		*Buttiauxella* spp., *n* = 1 (%)	0 (0)	0 (0)	0 (0)	0 (0)	0 (0)	0 (0)	0 (0)	0 (0)	0 (0)	0 (0)	0 (0)	0 (0)	0 (0)	0 (0)	0 (0)	**0 (0)**
		*Citrobacter* spp., *n* = 1 (%)	0 (0)	0 (0)	0 (0)	0 (0)	0 (0)	0 (0)	0 (0)	0 (0)	0 (0)	0 (0)	0 (0)	0 (0)	0 (0)	0 (0)	0 (0)	**0 (0)**
Brilliance™ VRE	Chicken (*n* = 13)	*Buttiauxella* spp., *n* = 2 (%)	1 (50)	0 (0)	1 (50)	0 (0)	0 (0)	0 (0)	0 (0)	0 (0)	1 (50)	0 (0)	0 (0)	0 (0)	0 (0)	2 (100)	0 (0)	**1 (50)**
		*Myroides* spp., *n* = 2 (%)	0 (0)	0 (0)	0 (0)	0 (0)	1 (50)	2 (100)	0 (0)	0 (0)	0 (0)	1 (50)	1 (50)	0 (0)	0 (0)	0 (0)	0 (0)	**0 (0)**
		*Serratia* spp., *n* = 9 (%)	6 (66.7)	5 (55.5)	3 (33.3)	1 (11.1)	1 (11.1)	0 (0)	0 (0)	0 (0)	3 (33.3)	0 (0)	0 (0)	0 (0)	1 (11.1)	1 (11.1)	1 (16.7)	**0 (0)**
	Pork (*n* = 4)	Serratia sp., *n* = 4 (%)	2 (50)	2 (50)	2 (50)	0 (0)	3 (75)	0 (0)	0 (0)	0 (0)	1 (25)	0 (0)	0 (0)	0 (0)	0 (0)	0 (0)	0 (0)	**0 (0)**
MAC/XLD	Chicken (*n* = 65)	*Aeromonas* spp., *n* = 3 (%)	0 (0)	0 (0)	0 (0)	0 (0)	0 (0)	0 (0)	0 (0)	0 (0)	0 (0)	0 (0)	0 (0)	0 (0)	1 (33.3)	0 (0)	0 (0)	**0 (0)**
		*Buttiauxella* spp., *n* = 2 (%)	1 (50)	1 (50)	0 (0)	0 (0)	0 (0)	0 (0)	0 (0)	0 (0)	1 (50)	0 (0)	0 (0)	0 (0)	0 (0)	0 (0)	0 (0)	**0 (0)**
		*Citrobacter* spp., *n* = 1 (%)	1 (100)	0 (0)	0 (0)	0 (0)	0 (0)	0 (0)	0 (0)	0 (0)	1 (100)	0 (0)	0 (0)	0 (0)	0 (0)	0 (0)	0 (0)	**0 (0)**
		*Enterobacter* spp., *n* = 2 (%)	1 (50)	1 (50)	0 (0)	0 (0)	0 (0)	0 (0)	0 (0)	0 (0)	1 (50)	0 (0)	0 (0)	0 (0)	0 (0)	0 (0)	0 (0)	**0 (0)**
		*Escherichia* spp., *n* = 46 (%)	2 (4.3)	3 (6.5)	46 (100)	0 (0)	46 (100)	0 (0)	0 (0)	0 (0)	0 (0)	0 (0)	0 (0)	0 (0)	1 (2.2)	6 (13)	0 (0)	**0 (0)**
		*Hafnia* spp., *n* = 8 (%)	2 (25)	0 (0)	6 (75)	0 (0)	6 (75)	6 (75)	0 (0)	0 (0)	6 (75)	6 (75)	0 (0)	0 (0)	6 (75)	6 (75)	0 (0)	**0 (0)**
		*Klebsiella* spp., *n* = 1 (%)	1 (100)	0 (0)	0 (0)	0 (0)	0 (0)	0 (0)	0 (0)	0 (0)	0 (0)	0 (0)	0 (0)	0 (0)	0 (0)	0 (0)	0 (0)	**0 (0)**
		*Serratia* spp., *n* = 2 (%)	1 (50)	0 (0)	0 (0)	1 (50)	1 (50)	1 (50)	0 (0)	1 (50)	1 (50)	1 (50)	1 (50)	0 (0)	2 (100)	2 (100)	0 (0)	**0 (0)**
	Pork (*n* = 26)	*Aeromonas* spp., *n* = 3 (%)	1 (33.3)	1 (33.3)	1 (33.3)	0 (0)	0 (0)	0 (0)	0 (0)	0 (0)	1 (33.3)	0 (0)	0 (0)	0 (0)	0 (0)	0 (0)	0 (0)	**0 (0)**
		*Buttiauxella* spp., *n* = 2 (%)	2 (100)	2 (100)	0 (0)	0 (0)	0 (0)	0 (0)	0 (0)	0 (0)	0 (0)	0 (0)	0 (0)	0 (0)	0 (0)	0 (0)	0 (0)	**0 (0)**
		*Citrobacter* spp., *n* = 1 (%)	1 (100)	1 (100)	0 (0)	0 (0)	0 (0)	0 (0)	0 (0)	0 (0)	1 (100)	0 (0)	0 (0)	0 (0)	0 (0)	0 (0)	0 (0)	**0 (0)**
		*Escherichia* spp., *n* = 6 (%)	1 (16.7)	0 (0)	0 (0)	0 (0)	0 (0)	0 (0)	0 (0)	0 (0)	0 (0)	0 (0)	0 (0)	0 (0)	0 (0)	0 (0)	0 (0)	**0 (0)**
		*Hafnia* spp., *n* = 14 (%)	3 (21.4)	4 (28.6)	0 (0)	0 (0)	0 (0)	0 (0)	0 (0)	0 (0)	0 (0)	0 (0)	0 (0)	0 (0)	0 (0)	0 (0)	0 (0)	**0 (0)**
CAM	Chicken (*n* = 7)	*Campylobacter* spp., *n* = 7 (%)	0 (0)	0 (0)	0 (0)	0 (0)	0 (0)	0 (0)	0 (0)	0 (0)	0 (0)	0 (0)	0 (0)	0 (0)	0 (0)	1 (16.7)	0 (0)	**0 (0)**

These data are pivotal for comprehending the antibiotic resistance profiles exhibited by bacterial isolates derived from the pooled chicken and pork samples. The table represents the AMR phenotype and MDR (35/288, 12%) for each isolate at the genus level. These isolates were cultured on selective media, including Brilliance™ ESBL, Brilliance™ CRE, Brilliance™ VRE agar, CAMPY, MAC, and XLD, from pooled chicken and pork samples. Specifically, for Gram-negative bacteria (CMV3AGNF Sensititre, Thermo Scientific™) 14 antibiotics comprising 9 CIAs and 5 HIAs, and for Campylobacter (EUCAMP2 Sensititre, Thermo Scientific™), 6 antibiotics comprising 5 CIAs and 1 HIA were tested against antibiotics at concentrations recommended by World Health Organization (2022). Additional details regarding selected antibiotics are provided in Supplementary Table S1.

Of the MDR isolates cultured on Brillance™ ESBL agar, *Serratia* spp. from chicken meat had the highest rate of MDR (15/56, 27%), which was significantly higher than *Serratia* spp. isolated from pork samples (2/30, 6.67%), although pooled chicken samples were more likely to be comprised of more than one product. Ciprofloxacin resistance was found in one of the MDR pork strains. One *Buttiauxella* spp. isolate from the chicken was resistant to four CIAs (AMP, AUG2, AXO, and FOX) and one HIA (CHL, SXT, and TET). One *Citrobacter* spp. isolate from the chicken was resistant to four CIAs (AMP, AUG2, AXO, and FOX) and one HIA (CHL), while the *Citrobacter* spp. isolate from pork was resistant to three CIAs (AMP, AUG2, and FOX) and two HIAs (SXT and TET). One chicken meat-derived *Enterobacter* spp. isolate was resistant to four CIAs (AMP, AUG2, AXO, and FOX) and three HIAs (CHL, SXT, and TET). One of the two MDR *Proteus* isolates from the chicken was resistant to two CIAs (AMP and AXO) and two HIAs (CHL, TET), while the other was resistant to four CIAs (AUG2, AXO, GEN, and STR) and one HIA (CHL). One *Rahnella* spp. and one *Yersinia* spp. isolate, both from pork meat, were resistant to three CIAs (AUG2, AMP, and AXO). One *Pseudomonas* spp. isolate from chicken meat and two strains from pork meat were resistant to multiple antibiotics; however, when the Magiorakos et al. (2012) definition of MDR was applied to *P. aeruginosa*, none of them were classified as MDR.

A total of nine isolates that grew on Brilliance™ CRE were MDR; eight were from chicken samples and one from a pork sample. Strains isolated from chicken samples belonged to the following genera: *Acinetobacter* (1/13, 7.7%), *Aeromonas* (2/3, 66.7%), *Enterobacter* (3/3, 100%), *E. coli* (1/1, 100%), and *Pseudomonas* (1/1, 100%). The MDR pork isolate was from *Aeromonas* spp. Several MDR isolates from Brilliance™ CRE were resistant to ciprofloxacin. One *Enterobacter* spp. isolate was highly MDR, as it was resistant to six CIAs (AUG2, AMP, FOX, AXO, CIP, and GEN) and one HIA (SXT), including ciprofloxacin. A strain of *E. coli* was resistant to five CIAs (AZI, AXO, CIP, NAL, and STR) and two HIAs (XNL and FIS), including ciprofloxacin. In addition, two *Acinetobacter* spp. isolates and one *Pseudomonas* spp. isolate were ciprofloxacin-resistant and MDR. Of the three *Enterobacter* spp. isolates from chicken meat that were MDR, one was resistant to ciprofloxacin. One *Aeromonas* spp. isolate from a pork sample was ciprofloxacin-resistant and MDR.

Of the 17 isolates that grew on Brilliance™ VRE agar, one *Buttiauxella* spp. isolate from a chicken sample displayed MDR. No isolates grown on MAC or XLD were MDR. Of the seven *Campylobacter* spp. isolates that grew on CAMPY agar, one displayed tetracycline resistance, but none were deemed MDR. No *Campylobacter* spp. were isolated from pork samples.

### Distribution of antimicrobial resistance genes

AMR genes were detected in the genomes of the 288 isolates using MobileElementFinder,^3^ a database for the identification of horizontally acquired AMR genes, virulence genes, and mobile genetic elements. Using a detection threshold of 95%, we found that 232/288 (81%) of the isolates carried at least one resistance gene (Table 3). AMR genes detected in these 232 isolates confer resistance to aminoglycosides, amphenicols, β-lactams, colistin, fosfomycin, hydrogen peroxide, olaquindox, quinolones, sulphonamides, tetracyclines, and trimethoprim. A full outline of the AMR genes for each strain is provided in Supplementary Table S1.

**Table 3:** Distribution of antimicrobial resistance genes found in bacterial isolates from pooled chicken and pork samples.

Antibiotics	Media	Genus	Resistance genes	Chicken (*n* = 206) Observed/total (%)	Pork (*n* = 82) Observed/total (%)
Aminoglycoside	Brilliance™ ESBL	*Proteus* spp.	*aadA1*	1/2 (50.0)	0/0 (0.0)
		*Pseudomonas* spp.	*aph(3′)-Ib*, *aph(6)-Id*	0/0 (0.0)	1/6 (16.7)
			*aph(3′)-llb*	5/28 (17.9)	1/6 (16.7)
	Brilliance™ CRE	*Stenotrophomonas* spp.	*aac(6′)-lz*, *aadA5*	1/7 (14.3)	0/0 (0.0)
			*aph(3′)-IIC*	3/7 (42.9)	0/0 (0.0)
	MAC/XLD	*Escherichia* spp.	*aadA1*	0/0 (0.0)	1/6 (16.7)
			*aph(3″)-lb*, *aph(6)-Id*	3/46 (6.5)	0/0 (0.0)
Amphenicols	Brilliance™ ESBL	*Pseudomonas* spp.	*catB7*	5/28 (17.9)	1/6 (16.7)
	MAC/XLD	*Escherichia* spp.	*catB7*	0/0 (0.0)	1/6 (16.7)
β-lactamase	Brilliance™ ESBL	*Achromobacter* spp.	*blaL1*	1/2 (50.0)	0/0 (0.0)
		*Aeromonas* spp.	*ampS*, *cphA4*	0/0 (0.0)	1/1 (100.0)
		*Buttiauxella* spp.	*qacE*	1/1 (100.0)	0/0 (0.0)
		*Citrobacter* spp.	*blaCMY-101*	0/0 (0.0)	1/1 (100.0)
			*blaCMY-82*	1/1 (100.0)	0/0 (0.0)
		*Pseudomonas* spp.	*blaOXA-485*, *blaOXA-488*	0/0 (0.0)	1/6 (16.7)
			*blaOXA-494*	2/28 (7.15)	0/0 (0.0)
			*POM-1*	2/28 (7.15)	3/6 (50.0)
			*qacE*	3/28 (10.7)	1/31 (3.2)
			*blaOXA-50*, *blaOXA-396*	5/28 (17.9)	0/0 (0.0)
			*blaPAO*	5/28 (17.9)	1/6 (16.7)
		*Rahnella* spp.	*blaRAHN-2*	0/0 (0.0)	1/1 (100.0)
		*Serratia* spp.	*blaFONA-1*	0/0 (0.0)	2/31 (6.4)
			*blaFONA-4*	1/56 (1.8)	1/31 (3.2)
			*blaFONA-2*	1/56 (1.8)	3/31 (9.7)
			*blaFONA-5*	3/56 (5.3)	5/31 (16.1)
			*blaFONA-6*	50/56 (89.3)	19/31 (61.3)
		*Yersinia* spp.	*blaFONA-6*	0/0 (0.0)	1/1 (100.0)
	Brilliance™ CRE	*Acinetobacter* spp.	*blaOXA-67*	0/0 (0.0)	4/8 (50)
			*blaMUS-1*, *blaOXA-117*, *blaOXA-120*, *blaOXA-355*, *blaOXA-98*	1/13 (7.7)	0/0 (0.0)
			*blaOXA-64*	2/13 (15.4)	0/0 (0.0)
			*blaOXA-51*	4/13 (30.8)	0/0 (0.0)
			*blaADC-25*	9/13 (69.2)	4/8 (50)
		*Aeromonas* spp.	*blaFONA-1*	0/0 (0.0)	1/1 (100)
			*ampS*, *blaCEPH-A3*, *blaFONA-2*, *blaRAHN-2*	1/3 (33.3)	0/0 (0.0)
			*cphA5*	1/3 (33.3)	1/1 (100)
		*Citrobacter* spp.	*blaACC-3*	0/0 (0.0)	1/1 (100)
		*Enterobacter* spp.	*blaACT-4*	2/3 (66.7)	0/0 (0.0)
		*Myroides* spp.	*blaMUS-1*	2/2 (100)	0/0 (0.0)
		*Stenotrophomonas* spp.	*blaL1*, *qacE*	1/7 (14.3)	0/0 (0.0)
	Brilliance™ VRE	*Buttiauxella* spp.	*blaFONA-6*	1/2 (50)	0/0 (0.0)
		*Serratia* spp.	*blaFONA-5*	2/9 (22.2)	0/0 (0.0)
			*blaFONA-6*	9/9 (100)	1/4 (25)
	MAC/XLD	*Aeromonas* spp.	*ampS*, *blaTEM-1B*	0/0 (0.0)	1/3 (33.3)
			*cphA4*	2/3 (66.7)	0/0 (0.0)
		*Citrobacter* spp.	*blaCMY-89*	1/1 (100)	0/0 (0.0)
		*Escherichia* spp.	*blaSHV-56*, *qacE*, *blaTEM-1B*	0/0 (0.0)	1/6 (16.7)
			*cphA5*	1/46 (2.2)	0/0 (0.0)
		*Hafnia* spp.	*blaACC-3*	0/0 (0.0)	1/14 (7.1)
			*blaACC-1*	1/8 (12.5)	0/0 (0.0)
			*blaACC-1a*	1/8 (12.5)	11/14 (78.6)
			*blaACC-5*, *blaCMY-105*	2/8 (25)	0/0 (0.0)
			*blaACC-1b*	6/8 (75.0)	1/14 (7.1)
		*Klebsiella* spp.	*blaSHV-40*, *blaSHV-56*, *blaSHV-79*, *blaSHV-85*, *blaSHV-89*	1/1 (100)	0/0 (0.0)
		*Serratia* spp.	*blaFONA-6*	2/2 (100)	0/0 (0.0)
	CAMPY	*Campylobacter* spp.	*blaOXA-193*, *blaOXA-450*, *blaOXA-451*, *blaOXA-452*, *blaOXA-453*, *blaOXA-489*, *blaOXA-61*, *blaTEM-116*	4/7 (66.7)	0/0 (0.0)
Colistin	Brilliance™ ESBL	*Achromobacter* spp.	*mcr-5.1*	1/1 (100.0)	0/0 (0.0)
		*Serratia* spp.	*mcr-9*	0/0 (0.0)	1/31 (3.2)
	MAC/XLD	*Aeromonas* spp.	*mcr-3.15*	1/3 (33.3)	0/0 (0.0)
Formaldehyde	MAC/XLD	*Escherichia* spp.	*formA*	1/46 (2.2)	0/0 (0.0)
Fosfomycin	Brilliance™ ESBL	*Pseudomonas* spp.	*fosA*	5/28 (17.9)	1/6 (16.7)
	Brilliance™ CRE	*Enterobacter* spp.	*fosA*	2/3 (66.6)	0/0 (0.0)
	MAC/XLD	*Klebsiella* spp.	*fosA*	1/1 (100)	0/0 (0.0)
Hydrogen peroxide	Brilliance™ CRE	*Escherichia* spp.	*sitABCD*	1/1 (100)	0/0 (0.0)
	Brilliance™ VRE	*Myroides* spp.	*sitABCD*	2/2 (100)	0/0 (0.0)
	MAC/XLD	*Escherichia* spp.	sitABCD	28/46 (60.9)	2/6 (33.3)
Olaquindox	MAC/XLD	*Klebsiella* spp.	*OqxA*, *OqxB*	1/1 (100)	0/0 (0.0)
Quinolones	Brilliance™ ESBL	*Citrobacter* spp.	*qnrB72*	1/1 (100.0)	0/0 (0.0)
	MAC/XLD	*Citrobacter* spp.	*qnrB60*	1/1 (100)	0/0 (0.0)
Sulphonamide	Brilliance™ ESBL	*Pseudomonas* spp.	*sul1*	0/0 (0.0)	1/6 (16.7)
			*crpP*	3/28 (10.7)	1/6 (16.7)
	Brilliance™ CRE	*Stenotrophomonas* spp.	*sul1*	1/7 (14.3)	0/0 (0.0)
	MAC/XLD	*Aeromonas* spp.	*sul1*	1/3 (33.3)	0/0 (0.0)
		*Escherichia* spp.	*sul2*	3/46 (6.5)	0/0 (0.0)
Tetracycline	Brilliance™ ESBL	*Aeromonas* spp.	*tet(E)*	0/0 (0.0)	1/6 (16.7)
	Brilliance™ CRE	*Aeromonas* spp.	*tet(E)*	1/3 (33.3)	0/0 (0.0)
		*Stenotrophomonas* spp.	*tet(A)*	1/7 (14.3)	0/0 (0.0)
	Brilliance™ VRE	*Serratia* spp.	*tet(A)*	1/9 (11.1)	2/4 (50)
	MAC/XLD	*Aeromonas* spp.	*tet(E)*	1/3 (33.3)	1/3 (33.3)
		*Escherichia* spp.	*tet(A)*	7/46 (15.2)	2/6 (33.3)
	CAMPY	*Campylobacter* spp.	*tet(O)*	1/7 (16.6)	0/0 (0.0)
Trimethoprim	MAC/XLD	*Escherichia* spp.	*dfrA5*, *dfrA14*	1/46 (2.2)	0/0 (0.0)

These data represent the antibiotic resistance genes detected in bacterial isolates, categorising them across 12 distinct antibiotic classes. Notably, during AST, five of these classes were scrutinised. Among these, three were CIAs: aminoglycoside, β-lactamase, and quinolone, while two were HIAs: sulphonamide and tetracycline.

### Aminoglycoside resistance genes

A total of 6/91 (7%) isolates from Brilliance™ ESBL agar from chicken meat and 1/41 (2%) from pork were found to contain aminoglycoside resistance genes. Among the chicken isolates, one *Proteus* spp. isolate (1/2, 50%) carried the *aadA1* gene, and 5/28 (18%) of *Pseudomonas* spp. carried the *aph(3′)-IIb* gene. One (17%) pork *Pseudomonas* spp. isolate carried the *aph(3′)-Ib* gene. A total of four (13%) chicken isolates from Brilliance™ CRE agar harboured aminoglycoside resistance genes: two *Stenotrophomonas* spp. isolates carried *aph(3′)-IIC*, one carried *aph(3′)-IIC* and *aac(6′)-Iz*, and another carried *aph(3′)-IIC* and *aadA5*. None of the pork isolates from Brilliance™ CRE agar nor any isolates from Brilliance™ VRE agar (both chicken and pork) carried aminoglycoside resistance genes. Of the isolates that grew on MAC, 3/46 (7%) *Escherichia* spp. isolates from chicken carried both *aph(3″)-Ib* and *aph(6)-Id*, and one isolate from pork carried the *aadA1* gene.

### β-lactamase resistance genes

Of the isolates obtained from Brilliance™ ESBL agar, 59/91 (65%) from chicken and 26/41 (63%) from pork harboured genes conferring resistance to β-lactam antibiotics. Of the chicken isolates, one *Achromobacter* spp. carried *blaL1*, one *Buttiauxella* spp. carried *qacE*, and one *Citrobacter* spp. carried *blaCMY-82*. A total of 11/28 (39%) *Pseudomonas* spp. carried β-lactamase resistance genes; two carried multiple genes (*blaOXA-494*, *blaOXA-50*, *blaOXA-396*, and *blaPAO*); another two carried only *POM-1*; three carried *blaOXA-50* and *blaPAO*; and four isolates carried only *qacE*. Of the *Serratia* spp. isolates, 55/56 (98%) carried a *blaFONA* gene variant, with variant *blaFONA-6* being the most common (*n* = 50). Among the pork isolates, one *Aeromonas* spp. carried *ampS* and *cphA4*, one *Citrobacter* spp. carried *blaCMY-101*, three *Pseudomonas* spp. carried *POM-1* only, and another *Pseudomonas* spp. isolate carried *blaPAO*, *blaOXA-485*, and *blaOXA-488*. One *Rahnella* spp. carried *blaRAHN-2*, and 30/31 (97%) *Serratia* spp. carried a *blaFONA* variant.

Of the isolates that grew on Brilliance™ CRE agar, 15/30 (50%) of the chicken isolates and 6/11 (55%) of the pork isolates carried a β-lactamase resistance gene. Among the chicken isolates, 11/13 (85%) *Acinetobacter* spp. carried various genes (*blaMUS-1*, *blaADC-25*, and *blaOXA* variants). Three *Aeromonas* spp., two *Enterobacter* spp., two *Myroides* spp., and one *Stenotrophomonas* spp. also carried various genes (*ampS*, *blaCEPH*, *balRAHN*, *cph*, *blaACT*, *blaMUS*, *blaL1*, *qacE*, and *blaFONA* variants). Among the pork isolates, four *Acinetobacter* spp., one *Aeromonas* spp., and one *Citrobacter* spp. carried β-lactamase resistance genes (*blaOXA*, *blaADC*, *cph*, *blaACC*, and *blaFONA-1*). Of the isolates that grew on Brilliance™ VRE agar, 12/13 (92%) of chicken and 3/4 (75%) of pork isolates carried β-lactamase resistance genes, with *blaFONA* variants being most common, particularly in *Serratia* spp.

Notably, isolates from MAC and XLD agar did not grow on Brilliance™ ESBL agar but were found to harbour β-lactamase resistance genes. These genes were identified in various species, including *Aeromonas* spp., *Citrobacter* spp., *Escherichia* spp., *Hafnia* spp., *Klebsiella* spp., and *Serratia* spp., in both chicken and pork samples. Of the *Campylobacter* isolates grown on CAMPY agar from chicken samples, 6/7 (86%, one *E. coli*, five *C. jejuni*) carried β-lactamase resistance genes, mostly *blaOXA* variants. One *C. jejuni* isolate harboured *blaTEM-116*.

### Quinolone resistance genes

A single *Citrobacter* spp. chicken isolate from Brilliance™ ESBL harboured *qnrB72*. Two *Enterobacter* spp. chicken isolates from Brilliance™ CRE carried *qnrE1*. No pork samples carried quinolone resistance genes. One *Citrobacter* spp. chicken isolate from MAC carried *qnrB60*. No pork isolates harboured a quinolone resistance gene.

### Sulphonamide resistance genes

Three chicken and one pork isolate from Brilliance™ ESBL agar harboured a sulphonamide resistance gene. All of these isolates were *Pseudomonas* spp., with chicken isolates carrying the *crpP* gene and the pork isolate carrying both *crpP* and *sul1*. One *Stenotrophomonas* spp. chicken isolate from Brilliance™ CRE agar carried *sul1*. Of the MAC isolates, one *Aeromonas* spp. carried *sul1*, and three *Escherichia* spp. carried *sul2*.

### Tetracycline resistance genes

A single *Aeromonas* spp. pork isolate from Brilliance™ ESBL carried *tet(E)*. Of the isolates obtained from Brilliance™ CRE agar, one *Aeromonas* spp. chicken isolate carried *tet(E)* and one *Stenotrophomonas* spp. carried *tet(A)*. Of the Brilliance™ VRE isolates, one *Serratia* spp. chicken isolate carried *tet(A)*, and two *Serratia* spp. pork isolates also harboured *tet(A)*. The majority of tetracycline resistance genes were detected in isolates from MAC/XLD agar. This included 7/46 (15%) *Escherichia* spp. from chicken samples and 2/6 (33%) *Escherichia* spp. from pork, which carried *tet(A)*. One *Aeromonas* spp. isolate from chicken and one from pork carried the *tet(E)* gene. A single strain of *C. jejuni* from CAMPY agar carried the *tet(O)*.

### Polymixin (colistin) resistance genes

Three isolates in this study were found to harbour a mobile colistin resistance (*mcr*) gene. One was a *Serratia* strain that was isolated on *Brilliance*™ ESBL agar and harboured the *mcr-9* variant. This strain also harboured the IncHI2 plasmid, which is known to be capable of carrying *mcr* genes, as well as a β-lactam resistance gene (*bla*FONA-6) and a gene conferring resistance to antiseptics (*qacE*). An *Aeromonas* strain, isolated from MacConkey agar, harboured the *mcr-3.15* variant and was detected in the same section of DNA (contig) as transposon Tn4671 and insertion sequence ISAs17, suggesting that these genetic elements may have played a role in the acquisition of *mcr-3.15*. This strain also harboured the tetracycline resistance gene, *tet(E)*. The third strain harbouring an *mcr* gene grew on *Brilliance*™ ESBL agar and was classified as *Achromobacter*. It harboured *mcr-5.1*, a contig containing the insertion sequence ISRme15, and *blaL1*.

### Resistance genes associated with other antimicrobials

Resistance genes associated with various other antimicrobials were identified. A fosfomycin resistance gene, *fosA*, was detected in eight different isolates, including five *Pseudomonas* spp., two *Enterobacter* spp., and one *Klebsiella* spp. A single *Escherichia* spp. isolate from MAC/XLD agar was identified as carrying the formaldehyde resistance gene, *formA*, and the hydrogen peroxide resistance and metal transporter gene *sitABCD* was present in 28/46 (61%) of *Escherichia* spp. A small number of strains from MAC/XLD carried genes conferring resistance to amphenicols, olaquindox, and trimethoprim.

### Distribution of plasmids

A large number of plasmids were detected across the 288 isolates (Supplementary Table S2). A list of plasmids (and resistance genes) associated with the MDR isolates is provided in Table 4.

**Table 4:** A list of resistance genes and plasmids detected in the multidrug-resistant isolates.

Bacterial genus/sp.	Meat type	Media	Antibiotic resistance gene(s)	Plasmid(s)
*Enterobacter* sp.	Chicken	CRE		
*Escherichia coli*	Chicken	CRE	*sit*ABCD	IncFIB(AP001918), IncFII
*Pseudomonas* sp.	Chicken	CRE		
*Acinetobacter baumanii*	Chicken	CRE	*bla*ADC-25, *bla*OXA-51	
*Enterobacter asburiae*	Chicken	CRE	*bla*ACT-4, *qnrE1*, *fosA*	
*Enterobacter asburiae*	Chicken	CRE	*bla*ACT-4, *qnrE1*, *fosA*	
*Aeromonas veronii*	Chicken	CRE	*ampS*, *bla*CEPH-A3, *tet(E)*	
*Aeromonas* sp.	Chicken	CRE	*ampS*, *bla*CEPH-A3, *tet(E)*	
*Aeromonas veronii*	Pork	CRE	*ampS*, *bla*CEPH-A4, *tet(E)*	
*Serratia* sp.	Pork	ESBL	*bla*FONA-6	IncHI1A, IncHI1B, ColE10
*Serratia* sp.	Pork	ESBL	*bla*FONA-6	ColE10
*Serratia* sp.	Chicken	ESBL	*bla*FONA-6	ColE10
*Serratia* sp.	Chicken	ESBL	*bla*FONA-6	
*Serratia* sp.	Chicken	ESBL	*bla*FONA-5	Col4401, ColE10
*Serratia* sp.	Chicken	ESBL	*bla*FONA-6	
*Serratia* sp.	Chicken	ESBL	*bla*FONA-2, *qacE*	
*Serratia* sp.	Chicken	ESBL	*bla*FONA-6	ColE10, ColE10
*Serratia* sp.	Chicken	ESBL	*bla*FONA-6	
*Serratia* sp.	Chicken	ESBL	*bla*FONA-6	ColE10, ColE10
*Serratia* sp.	Chicken	ESBL	*bla*FONA-6	ColE10, Col44011, ColRAAI, ColE10, IncN2
*Serratia* sp.	Chicken	ESBL		
*Serratia* sp.	Chicken	ESBL	*bla*FONA-6	
*Serratia* sp.	Chicken	ESBL	*bla*FONA-6	ColE10, ColE10
*Serratia* sp.	Chicken	ESBL	*bla*FONA-6	ColE10
*Serratia* sp.	Chicken	ESBL	*bla*FONA-6	
*Serratia* sp.	Chicken	ESBL	*bla*FONA-6	ColYe4449
*Buttiauxella* sp.	Chicken	ESBL	*qac*E	
*Buttiauxella* sp.	Pork	ESBL		
*Citrobacter braakii*	Pork	ESBL	*bla*CMY-101	Col(Ye4449)
*Citrobacter* sp.	Chicken	ESBL	*bla*CMY-82, *qnrB72*	IncFIB(pB171)
*Citrobacter* sp.	Chicken	ESBL	*bla*CMY-105, *bla*CMY-89, *qnrB60*	
*Enterobacter*	Chicken	ESBL		
*Proteus* sp.	Chicken	ESBL	*hug*A	
*Rahnella* sp.	Pork	ESBL	*bla*RAHN-2	
*Yersinia* sp.	Pork	ESBL	*bla*FONA-6	

Of the MDR isolates, 13/35 (37%) carried one or more plasmids. Nine of these isolates were *Serratia* spp., and all except one strain carried the ColE10 plasmid. Other plasmids in these strains included IncHI1A, IncHI1B, IncN2, Col4401, ColRAAI, and ColYe4449. One *Citrobacter* spp. carried the IncFIB(pB171) plasmid, and another *Citrobacter* spp. carried the Col(Ye4449) plasmid. One *Escherichia* spp. carried the IncFIB(AP001918) and IncFII plasmids.

### Distribution of virulence factors

One or more virulence genes were present in 61/288 (21%) of the isolates (Table 5). The vast majority of virulence factors were detected in *E. coli*, likely owing to the large number of virulence factors associated with this species in the VirulenceFinder database. All of the *E. coli* isolates in the study, apart from one isolate from pork, were found to harbour multiple virulence genes, ranging from 3 to 31 genes. Of note was the MDR *E. coli* isolate from Brilliance™ CRE agar, which had 17 virulence genes, nine of which were encoded on a plasmid (*etsC*, *iroN*, *cia*, *hlyF*, *cvaC*, *mchF*, *traT*, *ompT*, and *iss*), and the remainder elsewhere in the genome (*sitA*, *gad*, *terC*, *eilA*, *air*, *hlyE*, and *chuA*). All of the *E. coli* isolates (53/53, 100%) carried *terC*, consistent with other studies (Byarugaba et al., 2023). The next most frequent virulence factor was *traT* (37/46, 80%). Other common virulence factors included *chuA*, *cia*, *cvaC*, *etsC*, *fyuA*, *gad*, *hlyE*, *hlyF*, *hra*, *ipfA*, *ireA*, *iroN*, *irp2*, *iss*, *iucC*, *iutA*, *ompT*, and *sitA*.

**Table 5:** Distribution of virulence genes found in bacterial isolates from pooled chicken and pork samples.

Genus	Virulence genes	Chicken (*n* = 206) Observed/total (%)	Pork (*n* = 82) Observed/total (%)
Brilliance™ ESBL			
*Citrobacter* spp.	*traT*	1/1 (100)	1/1 (100)
*Serratia* spp.	*terC*	0/0 (0.0)	2/31 (6.45)
Brilliance™ CRE			
*Escherichia* spp.	*Air*, *chuA*, *cia*, *cvaC*, *eilA*, *etsC*, *gad*, *hylE*, *hlyF*, *iroN*, *iss*, *mchF*, *ompT*, *sitA*, *terC*, *traT*	1/1 (100)	
MAC/XLD			
*Citrobacter* spp.	*terC*	1/1 (100)	6/6 (100)
*Enterobacter* spp.	*terC*	2/2 (100)	0/0 (0.0)
*Escherichia* spp.	*afaA*, *afaB*, *afaC*, *afaE8*, *efa1*, *espJ*, *etpD*, *f17A*, *f17G*, *ibeA*, *mcbA*, *nleC*	1/46 (2.2)	0/0 (0.0)
	*papC*	1/46 (2.2)	1/6 (16.7)
	*Cib*, *kpsMII*, *papA_F48*	2/46 (4.3)	0/0 (0.0)
	*Cba*, *cif*, *eae*, *espA*, *espB*, *espF*, *nleB*, *papA_F11*, *tir*	3/46 (6.5)	0/0 (0.0)
	*afaD*, *celb*, *papA_F19*	4/46 (8.7)	0/0 (0.0)
	*nleA*	4/46 (8.7)	2/6 (33.3)
	*kpsMIII_K96*	5/46 (10.9)	0/0 (0.0)
	*Air*	7/46 (15.2)	2/6 (33.3)
	*eilA*, *iha*	8/46 (17.4)	2/6 (33.3)
	*pic*	9/46 (19.6)	0/0 (0.0)
	*astA*, *kpsMII_K1*	9/46 (19.6)	2/6 (33.3)
	*usp*	10/46 (21.7)	1/6 (16.7)
	*vat*	11/46 (23.9)	1/6 (16.7)
	*cma*	11/46 (23.9)	2/6 (33.3)
	*yfcV*, *cea*	12/46 (26.1)	1/6 (16.7)
	*tsh*	13/46 (28.3)	1/6 (16.7)
	*mchF*	14/46 (30.4)	3/6 (50)
	*neuC*	16/46 (34.8)	3/6 (50)
	*PacC*	17/46 (36.9)	3/6 (50)
	*kpsE*	18/46 (39.1)	1/6 (16.7)
	*fyuA*	20/46 (43.5)	0/0 (0.0)
	*irp2*	20/46 (43.5)	1/6 (16.7)
	*cia*, *ireA*, *ompT*	20/46 (43.5)	3/6 (50)
	*ipfA*	22/46 (47.8)	1/6 (16.7)
	*hra*	22/46 (47.8)	3/6 (50)
	*cvaC*	23/46 (50)	3/6 (50)
	*chuA*	26/46 (56.5)	3/6 (50)
	*iroN*	27/46 (58.7)	2/6 (33.3)
	*hlyF*, *iucC*, *iutA*	30/46 (65.2)	3/6 (50)
	*etsC*	31/46 (67.4)	1/6 (16.7)
	*gad*	33/46 (71.7)	6/6 (100)
	*sitA*	34/46 (73.9)	3/6 (50)
	*hlyE*	35/46 (76.1)	1/6 (16.7)
	*traT*	37/46 (80.4)	4/6 (66.7)
	*iss*	39/46 (84.8)	3/6 (50)
	*terC*	46/46 (100)	6/6 (100)
*Klebsiella* spp.	*iutA*, *traT*	1/1 (100)	0/0 (0.0)

### Pathogenicity of bacterial isolates

To determine the likelihood that an isolate is pathogenic to humans, the PathogenFinder tool from the CGE database was employed.^4^ Of the 288 bacterial isolates, 233 (81%) were determined to be pathogenic to humans, with probabilities ranging from 0.563 to 0.92. *Campylobacter* spp. and *Escherichia coli* exhibited notably high pathogenicity, with an average pathogenicity score of 0.91 and 0.87, respectively. In contrast, *Hafnia* spp. had the lowest pathogenicity, averaging 0.59. *Acinetobacter* spp. possessed the highest abundance of pathogenic protein families, totalling 402.2 on average. In contrast, *Hafnia* spp. exhibited the lowest number of pathogenic protein families, containing only 22.8 on average.

## Discussion

In this study, we describe the phenotypic and sequence-based AMR profiles of bacteria isolated from chicken and pork meat. Since only one representative for each colony morphology and colour was chosen from a limited number of selective agars, the total number and diversity of bacterial isolates present in the chicken and pork meat samples are likely to have been underestimated. Preferential selection of 288 isolates resulted in the detection of 17 bacterial genera or 33 bacterial species. Of the 288 isolates, 12% were MDR. By assessing the AMR phenotypic and genotypic profiles of species other than those typically used in surveillance studies, we were able to show that the reservoir of antimicrobial resistance to critically and highly important antibiotics in bacteria isolated from retail meat in Australia is more diverse than previously demonstrated.

In this study, we detected three mobilised colistin resistance (*mcr*) genes, conferring resistance to a last-line antimicrobial (colistin) for multidrug-resistant Gram-negative infections. These genes are capable of transmitting to different genera and species of bacteria that colonise humans and animals or are present in the environment. While Australia is thought to have a low abundance of colistin resistance, a recent analysis of host-derived and environmental metagenomes revealed that the highest log-ratio abundances of *mcr* fragments could be found in metagenomes from Australia when compared to countries all over the world (Martiny et al., 2022). This study showed that the *mcr*-9 variant is most common in Australia and that *mcr* variants are not equally distributed among different countries or bacterial genus/species. Surveillance studies of common foodborne pathogens would not have detected the presence of *mcr* variants in this study, as they were carried by bacteria not normally included in such studies, such as *Aeromonas*, *Achromobacter*, and *Serratia*.

The detection of multiple variants of *mcr* (variants −3.15, −5.1, and − 9) across different species of bacteria, harbouring other AMR genes on MGEs known to transmit *mcr* genes suggests that the distribution of *mcr* genes in Australia is likely to increase. Co-selection of *mcr* genes with AMR genes conferring resistance to antibiotics that are permitted for use in the agri-food industry in Australia, such as tetracycline, may accelerate the spread of colistin resistance. Although *Serratia* are known to be intrinsically resistant to colistin (Bean et al., 2020), we showed that *Serratia* are capable of acquiring *mcr* genes on plasmids that can be transferred to other species of bacteria. A ban on the prophylactic use of antibiotics, similar to that adopted by EU countries, may provide protection against the spread of colistin resistance. It is known that removing an antibiotic for use in the agri-food industry in Australia can result in a dramatic decline in resistance to that antibiotic. For example, a steady and significant reduction in erythromycin resistance in *Campylobacter* and *Enterococcus* isolated from food-producing chickens has been observed since the reduction in the use of macrolides in the 1990s, owing to the introduction of *Mycoplasma* vaccines (Australian Chicken Meat Federation, 2022). Similarly, the frequency of resistance to quinupristin-dalfopristin in *Enterococcus faecium* also significantly declined from 54.5 to 6.1% following a ban on the use of virginamycin in chickens in Australia. Removal of all antibiotics for prophylactic use in Australia would provide the best strategy for halting the spread of resistance to CIAs that arises due to the co-selection of genes conferring antimicrobial resistance to antibiotics currently used for “prevention” and genes conferring resistance to CIAs on MGEs.

The majority of the *Enterobacteriaceae* identified in this study belonged to the genus *Serratia*. Members of this genus were found to have acquired resistance genes for aminoglycosides, β-lactamases, fosfomycin, quinolones, amphenicols, and polypeptides, in a study by Sandner-Miranda et al. (2018). *Serratia* spp. isolates in this study harboured acquired resistance genes for colistin, β-lactamases, fosfomycin, and quinolones. *Serratia marcesensi*, which was previously thought to be a non-pathogenic environmental species, is responsible for a number of hospital-acquired infections, and MDR is already making infections with this species difficult to treat (Moradigaravand et al., 2016). It is largely unknown if other species of *Serratia* are contributing to AMR in *S. marcesens* and if AMR in the wider species poses a threat to human health as opportunistic pathogens.

The vast majority of virulence genes identified in this study were in *E. coli* isolates. However, genomic data for *E. coli* are more common than some of the lesser-known species identified, and therefore the virulence database is likely biased towards detecting virulence genes associated with *E. coli*. Several studies have shown that APEC and ExPEC virulence genes share similarities. Virulence genes such as *iss*, *iuA*, *ompT*, *papGII*, and *sfa* have been detected in zoonotic pathogens (Najafi et al., 2019). In this study, *papA*, *papC*, *usp., kpsMII*, and *ibeA* were frequently detected in *E. coli*, similar to APEC isolates harbouring *pap*, *sfa*, *usp., cnf1*, *kpsMTII*, *hlyA*, and *ibeA* virulence genes (Cunha et al., 2017). ExPEC-related virulence genes include *astA*, *cvaC*, *hra*, *hlyF*, *fyuA*, *ibeA*, *ireA*, *iss*, *ompT*, *papA*, *papC*, *papE*, *papF*, *tsh*, and *traT*, all of which were detected in this study and were previously found to be prevalent in *E. coli* isolated from chicken meat samples (Mitchell et al., 2015). One study performed a cluster analysis of *E. coli* isolated from UTIs, community-dwelling humans, meat, and meat production animals, which included data on the presence of eight ExPEC-related virulence genes (*kpsM II*, *papA*, *papC*, *iutA*, *sfaS*, *focG*, *afa*, *hlyD*) and AMR and found a strong association between the isolates from the various sources, suggesting that strains isolated from meat and meat production animals may be zoonotic pathogens (Jakobsen et al., 2010). The findings of the current study suggest that *E. coli* isolates from chicken meat may pose a zoonotic risk to humans given that most of the isolates (98%) were predicted to be pathogenic towards humans and that 47/53 (89%) of the *E. coli* isolates were isolated from chicken meat.

The level of MDR in this study was not particularly high relative to other countries (Collignon, 2015). However, given that we used selective media containing antibiotics, this figure does not accurately reflect the level of MDR in bacteria isolated from chicken and pork meat in Australia. This approach did allow us, however, to observe the diversity of MDR bacteria that grew on the various selective media. While few species identified in this study are commonly associated with infections in humans, most have been associated with infections in humans. For the most part, the AMR genes detected using WGS did not explain the resistance phenotypes observed on the selective agars or in the commercial antibiotic plates. This may be due to not all resistance genes being discovered, particularly for the less-well-studied species identified in this study, or the resistance mechanisms being chromosomally encoded, rather than acquired. Nonetheless, we identified a variety and diversity of bacteria that harboured horizontally acquired genes conferring resistance to either critically or highly important antibiotics. Some of these were MDR and are likely to be pathogenic to humans.

AMR is a worldwide issue, and its management calls for a “One Health” approach. The use of antibiotics in food-producing animals maintains antibiotic resistance mechanisms that make treatment of resistant bacterial infections in humans and animals difficult. Future surveillance studies should include analysis of a greater diversity of bacteria, to ensure the full diversity of AMR genes is revealed.

## Data availability statement

The original contributions presented in the study are included in the article/Supplementary material, further inquiries can be directed to the corresponding author.

## Ethics statement

The article presents research on animals that do not require ethical approval for their study.

## Author contributions

OD: Data curation, Formal analysis, Methodology, Writing – original draft, Investigation. MB: Methodology, Writing – review & editing. AP: Methodology, Writing – review & editing, Investigation. CO’B: Methodology, Writing – review & editing, Conceptualization, Data curation, Formal analysis, Funding acquisition, Project administration, Resources, Supervision, Writing – original draft.

## Footnotes

^1^ https://eur-lex.europa.eu/eli/reg/2019/6/oj

^2^ https://poultryhealthtoday.com/nearly-60-of-us-broilers-now-raised-without-antibiotics-but-that-number-may-have-peaked/

^3^ https://cge.food.dtu.dk/services/MobileElementFinder/

^4^ https://cge.food.dtu.dk/services/PathogenFinder/
