# Supplementary material for: Diversity of antimicrobial-resistant bacteria isolated from Australian chicken and pork meat
Source: Front Microbiol. 2024 Feb 19;15:1347597. doi: 10.3389/fmicb.2024.1347597 (PMC10910072; doi:10.3389/fmicb.2024.1347597)
Supplement: Supplementary file 1 [file Table_1.pdf]

Supplementary Table S1 A list of critical and highly important antibiotics included in the Gram-negative (CMV3AGNF) and *Campylobacter* (UCAMP2) susceptibility testing

Antibiotic class	Gram-negative bacteria CMV3AGNF			*Campylobacter* UCAMP2		
	Antibiotic agents		Conc. (µg/ml)	Antibiotic agents		Conc. (µg/ml)
**β-lactam penicillin**	**AUG2**	Amoxicillin/Clavulanic acid	0.5/1-16/32			
**Penicillin**	**AMP**	Ampicillin	1-32			
**Macrolide**	**AZI**	Azithromycin	0.12-16	**ERY**	Erythromycin	1-128
**Broad-spectrum cephalosporin**	**AXO**	Ceftriaxone	0.25-64			
**Cephamycin**	**FOX**	Cefoxitin	0.5-32			
**Fluroquinolone**	**CIP**	Ciprofloxacin	0.015-4	**CIP**	Ciprofloxacin	0.015-4
**Aminoglycoside**	**GEN**	Gentamicin	0.25-16	**GEN**	Gentamicin	0.25-16
**Quinolone**	**NAL**	Nalidixic acid	0.5-32	**NAL**	Nalidixic acid	0.5-32
**Streptomycin**	**STR**	Streptomycin	2-64	**STR**	Streptomycin	2-64
**Cephalosporin**	**XNL**	Ceftiofur	0.12-8			
**Phenicol**	**CHL**	Chloramphenicol	2-32			
**Sulphonamide**	**FIS**	Sulfisoxazole	16-256			
**Tetracycline**	**TET**	Tetracycline	4-32	**TET**	Tetracycline	4-32
**Dihydrofolate reductase**	**SXT**	Trimethoprim/ sulfamethoxazole	0.12/2.38-4/76			

Antibiotics deemed critically (**black font**) or highly (**red font**) important for human health, according to the WHO (2022).
