# Supplementary material for: Diversity of antimicrobial-resistant bacteria isolated from Australian chicken and pork meat
Source: Front Microbiol. 2024 Feb 19;15:1347597. doi: 10.3389/fmicb.2024.1347597 (PMC10910072; doi:10.3389/fmicb.2024.1347597)
Supplement: Supplementary file 2 [file Table_2.pdf]

Supplementary Table S2 The table represents the distribution of plasmids found in bacterial isolates from pooled chicken and pork samples.

Genus	Plasmid(s)	Chicken (n=206) Observed/Total (%)	Pork (n=82) Observed/Total (%)
	**Brilliance™ ESBL**		
***Citrobacter* spp.**	Col(Ye4449)	0/0 (0.0)	1/ 1(100)
***Serratia* spp.**	IncHI1A(CIT), IncHI1B(CIT), IncHI2, IncHI2A	0/0 (0.0)	1/31 (3.2)
	IncR, IncX5	0/0 (0.0)	2/31 6.5)
	IncR	1/56 (1.8)	0/0 (0.0)
	IncN3	2/56 (3.6)	0/0 (0.0)
	Col(Ye4449)	3/56 (5.4)	0/0 (0.0)
	ColE10	20/56 (35.7)	11/31 (35.5)
	**Brilliance™ CRE**		
***Escherichia* spp.**	IncFIB(AP001918), IncFII	1/1 (100)	
	**Brilliance™ VRE**		
***Serratia* spp.**	IncX5	1/9 (11.1)	0/0 (0.0)
	IncN3	2/9 (22.2)	0/0 (0.0)
	ColE10	3/9 (33.3)	1/4(25)
***Myroides* spp.**	rep6	1/2 (50)	0/0 (0.0)
	**MAC/XLD**		
***Citrobacter* spp.**	IncFII(Yp), RepA	0/0 (0.0)	1/1 (100)
	IncHI1A(CIT), IncHI1B(CIT), IncX5	1/1 (100)	0/0 (0.0)
***Enterobacter* spp.**	IncHI1A(CIT), IncHI1B(CIT)	2/2 (100)	0/0 (0.0)
***Escherichia* spp.**	IncFII(Yp)	0/0 (0.0)	1/6 (16.7)
	ColE440I, Col4401, IncFIA(HI1), IncFII(pCoo), IncFII(pHN7A8), IncFII(pRSB107), IncFII(pSE11), IncH1B(R27), IncX1, IncX4, IncY	1/46 (2.2)	0/0 (0.0)
	IncHI1B(R27)	1/46 (2.2)	1/6 (16.7)
	Col(BS512), IncFII(29), IncN	2/46 (4.3)	0/0 (0.0)
	IncHI1A	2/46 (4.3)	1/6 (16.7)
	IncHI2, IncL1	3/46 (6.5)	0/0 (0.0)
	Col(MG828)	3/46 (6.5)	1/6 (16.7)
	Col8282	3/46 (6.5)	2/6 (33.3)
	IncFIA, IncHI2A, IncI1	4/46 (8.7)	0/0 (0.0)
	Col156	5/46 (10.9)	1/6 (16.7)
	IncB/O/K/Z, IncI2	5/46 (10.9)	2/6 (33.3)
	p0111	7/46 (15.2)	0/0 (0.0)
	IncFIB(pLF82)	9/46 (19.6)	1/6 (16.7)
	IncFIC(FII)	15/46 (32.6)	0/0 (0.0)
	IncFII	16/46 (34.8)	4/6 (66.7)
	ColpVC	11/46 (23.9)	0/0 (0.0)
	IncFIB(AP001918)	31/46 (67.4)	4/6 (66.7)
	RepA	43/46 (93.5)	0/0 (0.0)
***Hafnia* spp.**	IncN3	0/0 (0.0)	1/14 (7.1)
***Klebsiella* spp.**	ColE440I, IncFIB(K), IncFII(K), IncN	1/1 (100)	0/0 (0.0)
***Serratia* spp.**	ColE10, IncN3	1/2 (50)	0/0 (0.0)
